# Proteasome inhibition induces IKK-dependent interleukin-8 expression in triple negative breast cancer cells: Opportunity for combination therapy

**DOI:** 10.1371/journal.pone.0201858

**Published:** 2018-08-08

**Authors:** Mohammad M. Uddin, Yue Zou, Tamanna Sharma, Himavanth R. Gatla, Ivana Vancurova

**Affiliations:** Department of Biological Sciences, St. John's University, New York, New York, United States of America; University of South Alabama Mitchell Cancer Institute, UNITED STATES

## Abstract

Triple negative breast cancer (TNBC) cells express increased levels of the pro-inflammatory and pro-angiogenic chemokine interleukin-8 (IL-8, CXCL8), which promotes their proliferation and migration. Because TNBC patients are unresponsive to current targeted therapies, new therapeutic strategies are urgently needed. While proteasome inhibition by bortezomib (BZ) or carfilzomib (CZ) has been effective in treating hematological malignancies, it has been less effective in solid tumors, including TNBC, but the mechanisms are incompletely understood. Here we report that proteasome inhibition significantly increases expression of IL-8, and its receptors CXCR1 and CXCR2, in TNBC cells. Suppression or neutralization of the BZ-induced IL-8 potentiates the BZ cytotoxic and anti-proliferative effect in TNBC cells. The IL-8 expression induced by proteasome inhibition in TNBC cells is mediated by IκB kinase (IKK), increased nuclear accumulation of p65 NFκB, and by IKK-dependent p65 recruitment to IL-8 promoter. Importantly, inhibition of IKK activity significantly decreases proliferation, migration, and invasion of BZ-treated TNBC cells. These data provide the first evidence demonstrating that proteasome inhibition increases the IL-8 signaling in TNBC cells, and suggesting that IKK inhibitors may increase effectiveness of proteasome inhibitors in treating TNBC.

## Introduction

Interleukin-8 (IL-8, CXCL8) is a pro-inflammatory and pro-angiogenic chemokine that stimulates cancer progression by inducing tumor cell proliferation, survival, and migration [1,2]. IL-8 expression is increased in many types of advanced cancers, including triple negative breast cancer (TNBC), and correlates with poor prognosis [3–6]. TNBC, characterized by the lack of estrogen (ER), progesterone (PR), and Her2 receptors, accounts for about 15–20% of all breast cancers, and is the subtype with the worst prognosis. Because no targeted therapies are currently available, and majority of TNBC patients initially responding to cytotoxic chemotherapy become drug-resistant, development of novel therapeutic strategies is essential [7]. Proteasome inhibition by bortezomib (BZ; Velcade; PS-341) and carfilzomib (CZ), developed for its ability to inhibit transcription of NFκB-dependent anti-apoptotic genes, has been effective in treating multiple myeloma and other hematological malignancies [8–11]. By contrast, as single agents, proteasome inhibitors (PI) have failed to show a significant clinical activity in solid tumors, including TNBC [12–17], but the responsible mechanisms are not fully understood.

IL-8 transcription is regulated by the transcription factor NFκB [18–20], which is constitutively activated in TNBC cells and tissues; inhibition of NFκB activity suppresses angiogenesis and tumorigenicity of TNBC cells [21–30]. Activation of NFκB is mediated by the enzymes of IκB kinase (IKK) complex, which phosphorylate the inhibitory protein IκBα, leading to its proteasomal degradation, nuclear translocation of NFκB subunits, and NFκB-dependent transcription [31–33]. However, in contrast to other NFκB-dependent genes that are regulated by p65/p50 NFκB heterodimers, the IL-8 transcription is regulated predominantly by p65 homodimers [19,34,35], making it particularly dependent on the mechanisms that regulate the nuclear p65 levels and p65 transcriptional activity [36]. Given that p65 can also undergo proteasomal degradation [37], proteasome inhibition can stabilize both IκBα and p65, thus potentially having two completely opposing effects on the regulation of NFκB-dependent genes. Indeed, previous studies from our laboratory have shown that while proteasome inhibition in cutaneous T cell lymphoma, prostate cancer, ovarian cancer, and monocytic cells suppresses transcription of genes regulated by p65/p50 NFκB heterodimers, it upregulates the p65 homodimer-dependent IL-8 transcription [38–41]. Interestingly, however, the induction of IL-8 expression by PI is cell specific; proteasome inhibition does not induce IL-8 expression in multiple myeloma cells [40], where PI exhibit significant clinical activity.

Since there are no effective therapies for TNBC, and the effect of PI on NFκB-dependent transcription in TNBC cells has never been investigated, in this study, we examined the effect of proteasome inhibition on the expression of NFκB-dependent genes in TNBC cells, and tested the hypothesis that proteasome inhibition induces IL-8 expression, resulting in increased proliferation and migration of TNBC cells. Our results are the first to show that proteasome inhibition in TNBC cells specifically upregulates expression of IL-8 and its receptors, CXCR1 and CXCR2. The induced IL-8 expression in TNBC cells is mediated by an increased nuclear accumulation of p65, and IKK-dependent p65 occupancy at the IL-8 promoter. Suppression or neutralization of the induced IL-8, or inhibition of IKK activity, enhances the BZ cytotoxic and anti-proliferative effect in TNBC cells, suggesting that by suppressing the IL-8 expression, IKK inhibitors may increase effectiveness of proteasome inhibitors in TNBC treatment.

## Materials and methods

### Antibodies and reagents

Antibodies against human CXCR1 (sc-7303), CXCR2 (sc-7304), IKKα (sc-7218), IKKβ (sc-8014), IKKε (sc-376114), p65 NFκB (sc-372), IκBα (sc-371), and histone H3 (sc-8654) were purchased from Santa Cruz Biotechnology (Santa Cruz, CA, USA). Antibody against lactate dehydrogenase (LDH; 20-LG22) was from Fitzgerald Industries International (North Acton, MA, USA), and actin antibody was from Sigma (St Louis, MO, USA). Horseradish peroxidase (HRP)-conjugated anti-rabbit and anti-mouse secondary antibodies were from Santa Cruz Biotechnology (Santa Cruz, CA).

Bortezomib was from ChemieTek (Indianapolis, IN, USA), and carfilzomib was from ApexBio (Houston, TX, USA). Bay-117082 was purchased from Sigma, and SC514 was from Santa Cruz Biotechnology. All other reagents were molecular biology grade and were from Sigma (St Louis, MO).

### Cell culture

All cell lines were obtained from American Type Culture Collection (ATCC, Manassas, VA, USA). Breast cancer MDA-MB-231, MDA-MB-468, and MCF-7 cells were cultured in Dulbecco's modified Eagle's medium (DMEM; ATCC, Manassas, VA) supplemented with 10% heat inactivated fetal bovine serum (FBS; Invitrogen, Grand Island, NY, USA) and antibiotics (100 units/ml penicillin and 100 μg/ml streptomycin). HCC-1937 cells were cultured in RPMI 1640 medium (Invitrogen) supplemented with 2 mM L-glutamine, 10 mM HEPES, 1 mM sodium pyruvate, 10% heat inactivated FBS, and antibiotics (100 units/ml penicillin and 100 μg/ml streptomycin). Before treatment, cells were seeded (5 × 10^5^ cells/ml) for 24 h in 6-well plates and grown at 37°C with 5% CO_2_. Bortezomib, carfilzomib, and IKK inhibitors Bay-117082 and SC-514 were dissolved in DMSO and stored at −80°C. An equivalent volume of DMSO was used in all experiments as a solvent control. Cell viability was measured by using Trypan Blue exclusion.

### Transfection with siRNA

Human IL-8 (sc-39631), IKKα (sc-29365), IKKβ (sc-35644), IKKε (sc-39056), and non-silencing (sc-37007) small interfering RNAs (siRNAs) were obtained from Santa Cruz Biotechnology. Prior to transfection, cells were seeded (250,000 cells/ml) into a 6-well plate and incubated in a humidified 5% CO_2_ atmosphere at 37°C in antibiotic-free RPMI medium supplement with 10% FBS for 24 h to about 80% confluence. For each transfection, 80 pmol of either non-silencing siRNA control or specific siRNA were used; cells were transfected 7 h in siRNA transfection medium with siRNA transfection reagent according to manufacturer’s instructions (Santa Cruz Biotechnology). After transfection, fresh medium with antibiotics was added, and cells were grown for 24 h before treatment.

### Cell proliferation and IL-8 neutralizing assays

Cell proliferation was measured by CellTiter 96 One Solution Cell Proliferation Assay (Promega, Madison, WI, USA). Cells were seeded into 96-well plates at a density of 5000 cells/100 μl of medium, and incubated with BZ, CZ, or control DMSO at 37°C. At indicated time points, 20 μl of CellTiter 96 One Solution Reagent was added to each well, incubated for 4 h at 37°C, and absorbance at 490 nm was measured.

For IL-8 neutralization experiments, MDA-MB-231 cells were incubated 24 h with BZ in the presence of 2 μg/ml of anti-human IL-8 monoclonal IgG1 antibody (MAB208; R&D, Minneapolis, MN, USA) or control mouse IgG1 (MAB002; R&D), and cell viability and proliferation were measured as described above.

### Cell invasion and wound-healing scratch assays

Invasion assay was performed as described by the manufacturer’s protocol (Corning, NY, USA). MDA-MB-231 cells were seeded onto the Corning Biocoat Matrigel Chambers (Corning #354480) at a density of 25,000 cells/0.5 ml in serum-free DMEM medium, with bottom wells containing DMEM medium with 10% FBS. Cells in the top chambers were treated with 10 μM Bay-117082 for 12 h, followed by 10 nM BZ for 12 h. After incubation, non-invading cells were scrubbed from the upper surface of the top chambers using cotton swabs. Matrigel invading cells on the bottom membranes were fixed with 100% methanol, stained with 0.5% crystal violet, washed, and air dried. Invading cells were counted in five randomly selected fields under a phase-contrast microscope at 20X magnification, and quantified using ImageJ software.

For wound-healing scratch assay, MDA-MB-231 cells were seeded in 6-well plates at a density of 500,000 cells/ml of medium containing 5% FBS. Once cells reached 90% confluency, they were incubated 12 h with 10 μM Bay-117082 or control DMSO, scratched using a sterile 200 μl pipette tip, and incubated another 24 h with 10 nM BZ. The scratch area was monitored under phase-contrast microscope at 0 and 24 h after BZ treatment. Scratch width was measured in 5 randomly selected areas at 10X magnification using ImageJ software.

Supernatants from cell invasion and wound-healing assays were collected for IL-8 analysis by ELISA.

### Real time PCR

Total RNA was isolated by using RNeasy mini-kit (Qiagen, Valencia, CA, USA). The iScript one-step RT-PCR kit with SYBR Green (Bio-Rad, Hercules, CA, USA) was used as a supermix and 20 ng/μl of RNA was used as template on a Bio-Rad MyIQ Single Color Real-Time PCR Detection System (Bio-Rad). The primers used for quantification of human IL-8, cIAP-1, cIAP-2, Bcl2, Bcl-xL, PD-L1, CXCR1, CXCR2, and actin mRNA were purchased from SA Biosciences (Frederick, MD, USA). The mRNA values are expressed as a percentage of untreated (UT) samples, which were arbitrarily set as 100%.

### ELISA

IL-8 release was measured in cell supernatants by commercially available IL-8 ELISA kit (D8000C; R&D, Minneapolis, MN).

### Western blotting

Whole cell extracts (WCE), and nuclear (NE) and cytoplasmic extracts (CE) were prepared as described previously [42], and separated on 12% SDS gels. To determine equal protein loading, membranes were stripped and re-probed with anti-actin antibody. Contamination of nuclear and cytoplasmic fractions by cytoplasmic and nuclear proteins, respectively, was determined by immunoblotting using lactate dehydrogenase (LDH) and histone H3 as specific markers, as described [42].

### Chromatin immunoprecipitation (ChIP)

ChIP analysis was performed as described [43]. The promoter occupancy was calculated by using the human IGX1A negative control primers (SA Biosciences, Frederick, MD), which detect specific genomic ORF-free DNA sequence that does not contain binding site for any known transcription factors. The results were calculated as fold difference in p65 occupancy at the IL-8 promoter in comparison with the IGX1A locus. The IL-8 primers used for real time PCR were the following: forward, 5'-GGGCCATCAGTTGCAAATC-3' and reverse, 5’-GCTTGTGTGCTCTGCTGTCTC-3'.

### Statistical analysis

The results represent at least three independent experiments. Numerical results are presented as means ± SE. Data were analyzed by using an InStat software package (GraphPAD, San Diego, CA, USA). Statistical significance was evaluated by using Mann-Whitney U test, and p<0.05 was considered significant. Levels of significance are indicated as *p<0.05; **p<0.01; and ***p<0.001.

## Results

### Proteasome inhibition upregulates IL-8 expression in TNBC cells

**Fig 1:**
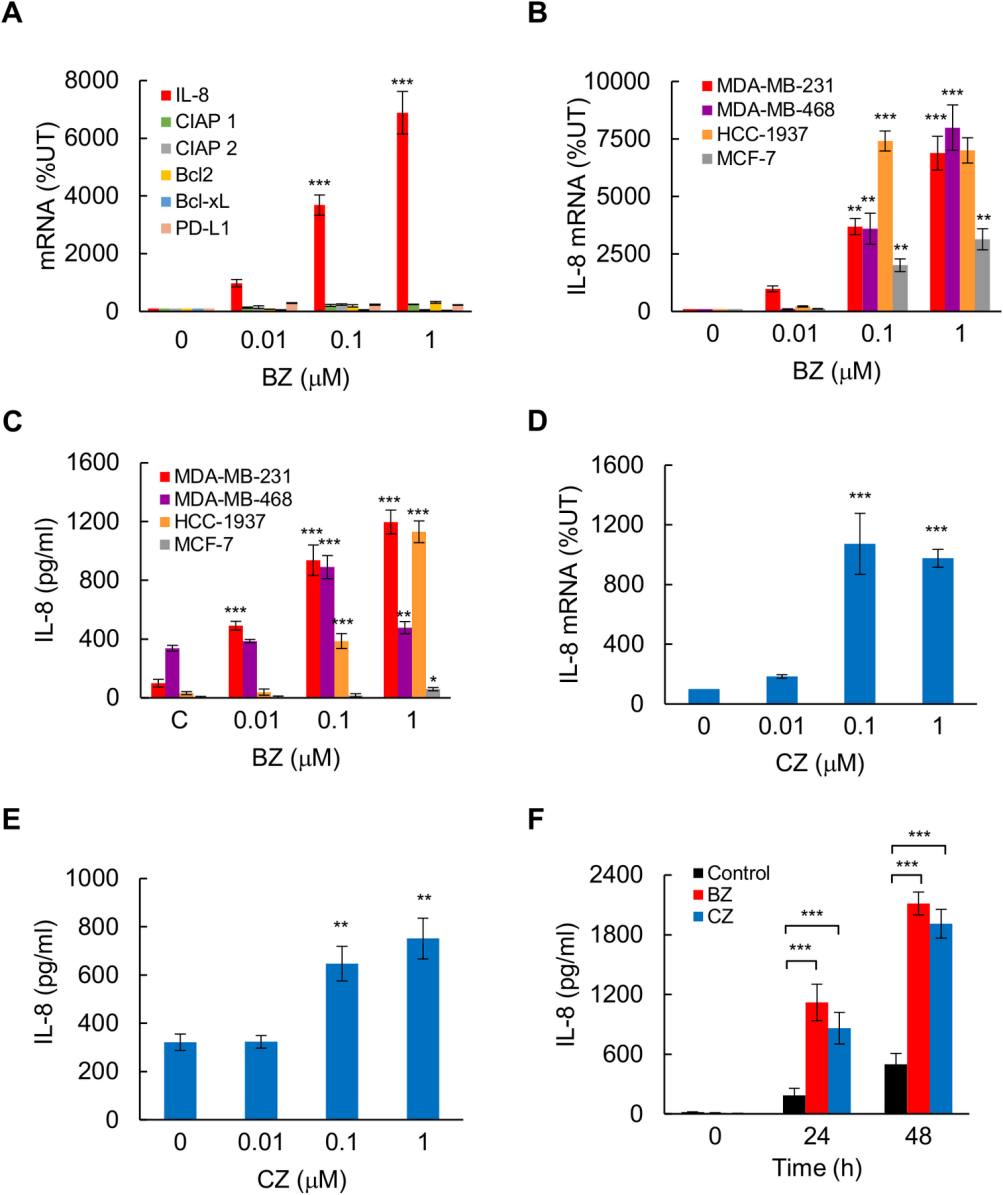
Proteasome inhibition upregulates IL-8 expression in TNBC cells. **(A)** Real time RT-PCR of mRNA levels of different NFκB-dependent genes measured in MDA-MB-231 cells treated with increasing BZ concentrations for 24 hours. **(B)** RT-PCR of IL-8 mRNA in MDA-MB-231, MDA-MB-468, HCC-1937, and MCF-7 cells treated 24 h with increasing BZ. **(C)** IL-8 release measured by ELISA in cell culture supernatants of MDA-MB-231, MDA-MB-468, HCC-1937, and MCF-7 cells treated 24 h with increasing BZ. **(D)** RT-PCR of IL-8 mRNA in MDA-MB-231 cells treated 24 h with increasing CZ concentrations. **(E)** IL-8 release measured by ELISA in cell culture supernatants of MDA-MB-231 cells treated 24 h with increasing CZ. **(F)** IL-8 release in cell culture supernatants of MDA-MB-231 cells treated with control DMSO, 100 nM BZ, or 100 nM CZ for 0, 24, and 48 h. The values represent the mean +/− SE of four experiments; asterisks denote a statistically significant change compared to control untreated (UT) cells (* p<0.05; ** p<0.01; *** p<0.001).

To test the hypothesis that proteasome inhibition increases IL-8 expression in TNBC cells, we first analyzed expression of IL-8, as well as other NFκB-dependent genes, in bortezomib (BZ)-treated MDA-MB-231 cells that are characterized by the lack of ER, PR, and Her2 receptors, high proliferation rates and resistance to hormone therapy, and have been widely used as an *in vitro* model to study TNBC. Interestingly, while BZ did not have a significant effect on the expression of NFκB-regulated genes cIAP1, cIAP2, Bcl2, Bcl-xL, and PD-L1 in MDA-MB-231 cells, it dramatically increased the IL-8 mRNA levels (Fig 1A).

To determine whether PI induces the IL-8 expression also in other types of breast cancer cells, we analyzed IL-8 mRNA levels and cytokine release in BZ-treated MDA-MB-468, HCC-1937, and MCF-7 cell lines. Similar to the MDA-MB-231 cells, the MDA-MB-468 and HCC-1937 cell lines are ER, PR, and Her2-negative, and are unresponsive to hormone therapy. In contrast, the MCF-7 cells are ER and PR-positive, and are characterized by hormone sensitivity and lower proliferation rates. As shown in Fig 1B, 24 h incubation with 100 nM BZ, which approximately corresponds to the clinically used concentrations [44], significantly increased IL-8 mRNA levels in TNBC cells MDA-MB-468 and HCC-1937, as well as in ER/PR-positive MCF-7 cells. However, while 100 nM BZ greatly increased the IL-8 release in all three tested TNBC cell lines, the IL-8 release in MCF-7 cells was much lower (Fig 1C). Both IL-8 gene expression (Fig 1D) and cytokine release (Fig 1E) were induced in MDA-MB-231 cells also by carfilzomib (CZ), a second-generation proteasome inhibitor [45,46]. Incubation (24 and 48 h) of MDA-MB-231 cells with 100 nM BZ and CZ produced comparable levels of IL-8 release (Fig 1F).

### Suppression of BZ-induced IL-8 potentiates BZ cytotoxic and anti-proliferative effect in TNBC cells

**Fig 2:**
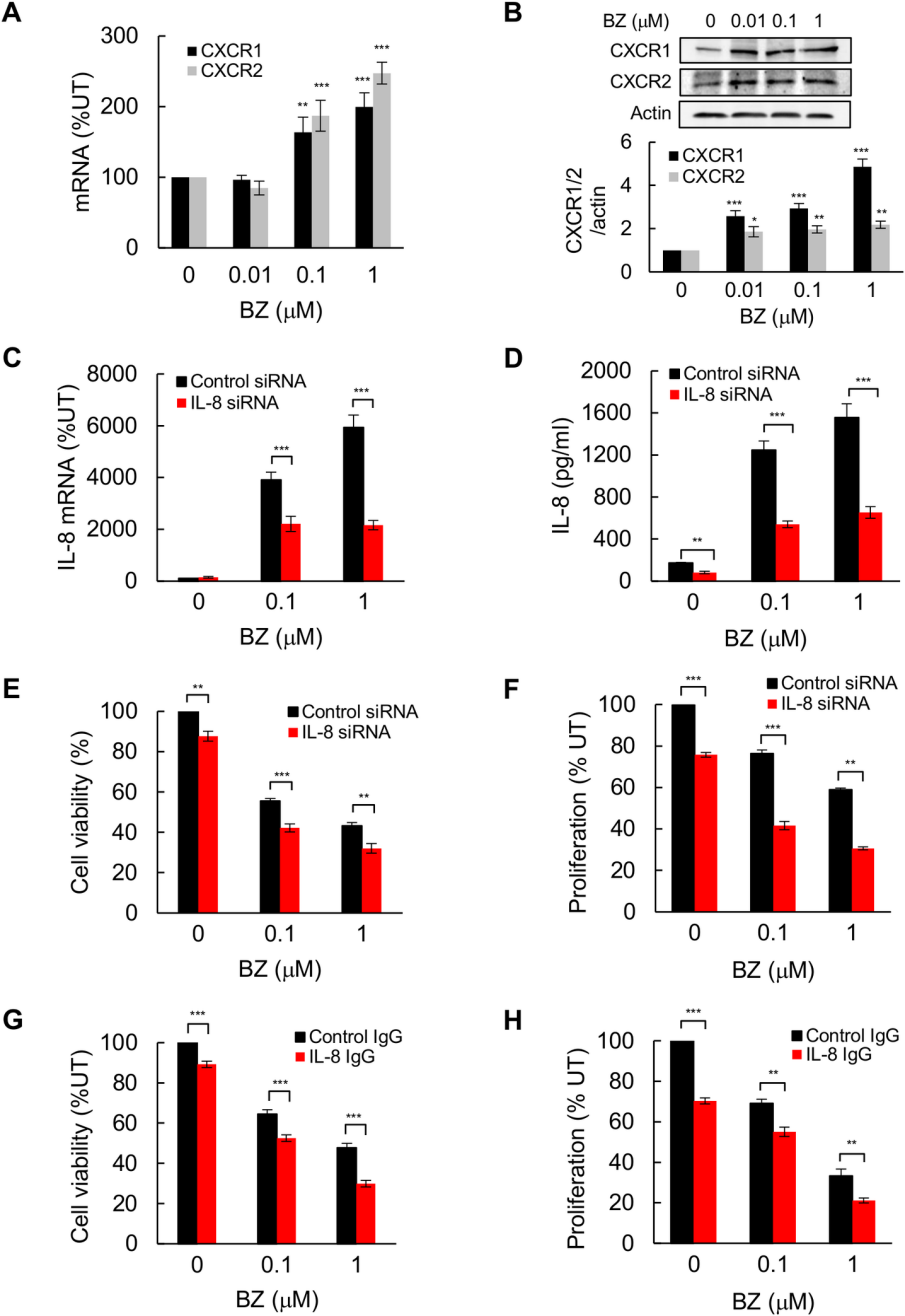
Suppression of BZ-induced IL-8 augments BZ cytotoxic and anti-proliferative effect in TNBC cells. **(A)** RT-PCR of CXCR1 and CXCR2 mRNA levels in MDA-MB-231 cells treated with increasing BZ for 24 h. **(B)** Western analysis of CXCR1, CXCR2, and control actin protein levels in whole cell extracts (WCE) of MDA-MB-231 cells treated with increasing BZ for 24 h. The bottom panel represents densitometric evaluation of CXCR1 and CXCR2 protein levels shown in the top panel. The CXCR1/2 densities were normalized to actin, and expressed relative to untreated cells. **(C)** RT-PCR of IL-8 mRNA in MDA-MB-231 cells transfected with control or IL-8 siRNA and incubated 24 h with increasing BZ. **(D)** IL-8 release measured by ELISA in supernatant of MDA-MB-231 cells transfected with control or IL-8 siRNA and incubated 24 h with increasing BZ. **(E)** Viability of MDA-MB-231 cells transfected with control or IL-8 siRNA, and incubated 24 h with increasing BZ, measured be Trypan Blue exclusion. **(F)** Proliferation of MDA-MB-231 cells transfected with control or IL-8 siRNA, and incubated 24 h with BZ, measured by CellTiter 96 One Solution Cell Proliferation Assay. **(G)** Viability of MDA-MB-231 cells incubated 24 h with increasing BZ in the presence of control pre-immune IgG or IL-8 neutralizing monoclonal antibody. **(H)** Proliferation of MDA-MB-231 cells incubated 24 h with increasing BZ in the presence of control pre-immune IgG or IL-8 neutralizing monoclonal antibody. The values represent the mean +/− SE of four experiments; asterisks denote a statistically significant change compared to control.

IL-8 mediates its functions through binding to its receptors, CXCR1 and CXCR2; both receptors are expressed in TNBC cells and are also regulated by NFκB [47,48]. To determine whether proteasome inhibition might regulate the expression of CXCR1 and CXCR2 in TNBC cells, we analyzed their gene and protein expression in BZ (24 h)-treated MDA-MB-231 cells. Interestingly, 100 nM BZ significantly increased both mRNA (Fig 2A) and protein (Fig 2B) levels of both receptors, indicating that proteasome inhibition increases IL-8 signaling in TNBC cells.

To determine whether suppression of the induced IL-8 expression might augment the BZ cytotoxic and anti-proliferative effect, we analyzed cell viability and proliferation in MDA-MB-231 cells transfected with IL-8 specific siRNA or control siRNA, and incubated 24 h with increasing BZ concentrations. IL-8 mRNA levels (Fig 2C) and cytokine release (Fig 2D) were significantly decreased in cells transfected with IL-8 specific siRNA compared to control siRNA. As previously observed [49,50], BZ decreased viability and proliferation of TNBC cells (Fig 2E and 2F). Importantly, IL-8 suppression significantly potentiated the BZ-mediated cytotoxic effect (Fig 2E). Furthermore, IL-8 suppression also significantly decreased cell proliferation in both untreated and BZ-treated MDA-MB-231 cells (Fig 2F). To validate the above results and determine whether they can be duplicated by neutralization of the produced IL-8, we analyzed viability and proliferation of MDA-MB-231 cells incubated 24 h with BZ in the presence of IL-8 neutralizing antibody. As shown in Fig 2G, compared to control mouse IgG1, incubation with mouse anti-IL-8 IgG1 significantly decreased viability of untreated and BZ-treated MDA-MB-231 cells. In addition, IL-8 neutralization significantly reduced proliferation of untreated and BZ-treated MDA-MB-231 cells (Fig 2H), indicating that inhibition of the BZ-induced IL-8 expression enhances BZ cytotoxic and anti-proliferative effect in TNBC cells.

### BZ-induced IL-8 expression in TNBC cells is mediated by IKK

**Fig 3:**
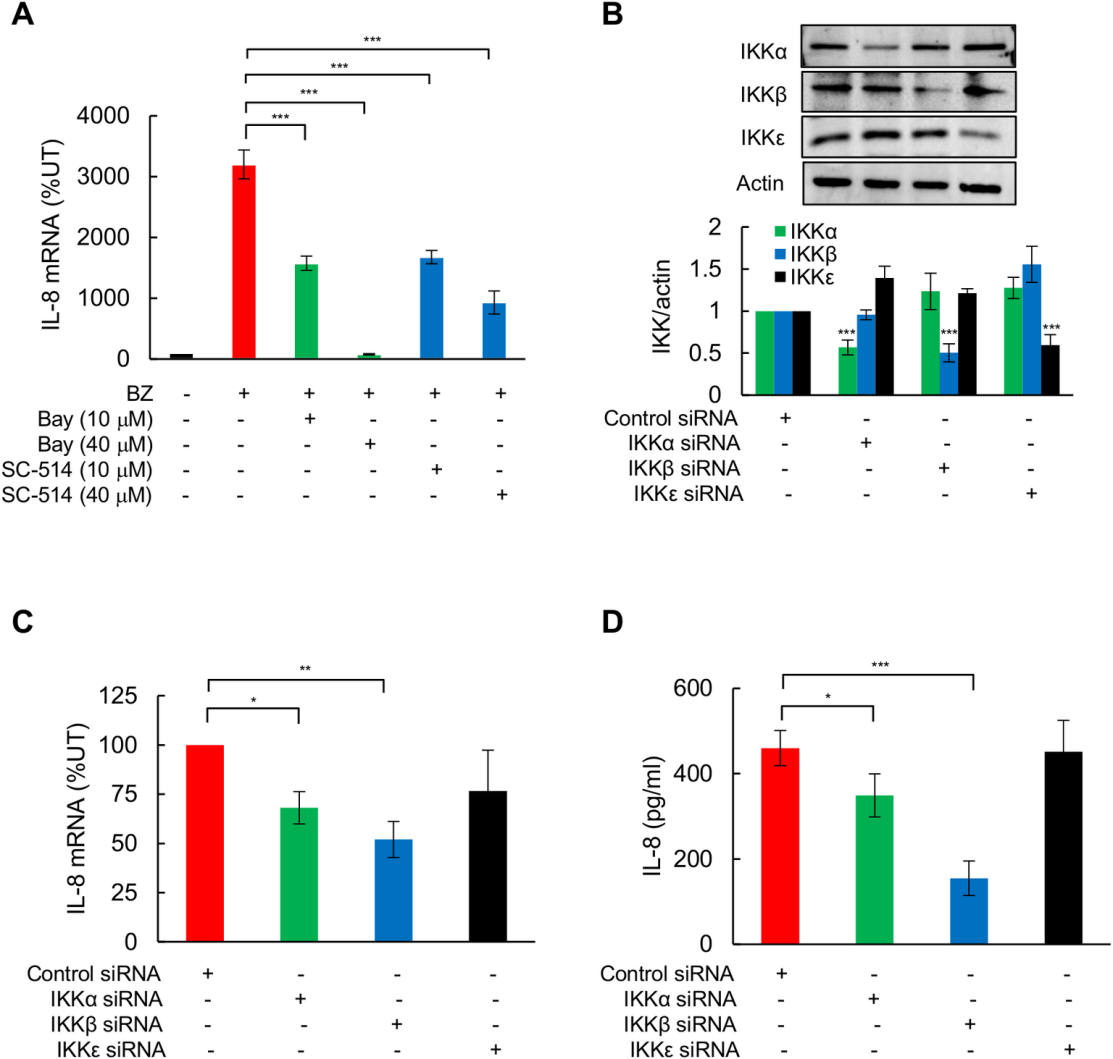
BZ-induced IL-8 expression in TNBC cells is mediated by IKK. **(A**) RT-PCR of IL-8 mRNA in MDA-MB-231 cells pre-treated 12 h with control DMSO, Bay-117082 (10 or 40 μM), or SC-514 (10 or 40 μM), and incubated 24 h with 100 nM BZ. **(B)** Western analysis of IKKα, IKKβ, and IKKε in WCE of MDA-MB-231 cells transfected with control, IKKα, IKKβ, and IKKε siRNA, and treated with BZ (100 nM, 24 h). The bottom panel represents densitometric evaluation of IKKα, IKKβ, and IKKε protein levels shown in the top panel; the IKKs densities were normalized to actin, and expressed relative to cells transfected with control siRNA. **(C)** RT-PCR of IL-8 mRNA in MDA-MB-231 cells transfected with control, IKKα, IKKβ, or IKKε siRNA, and incubated 24 h with 100 nM BZ. **(D)** IL-8 release measured by ELISA in MDA-MB-231 cells transfected with control, IKKα, IKKβ, or IKKε siRNA and incubated 24 h with 100 nM BZ. The values represent the mean +/− SE of four experiments; asterisks denote a statistically significant change compared to control.

To understand the mechanism of how proteasome inhibition increases IL-8 expression in TNBC cells, we first investigated IKK involvement in the BZ-induced IL-8 expression, and analyzed IL-8 mRNA levels in BZ-treated MDA-MB-231 cells pre-incubated with a broad-spectrum inhibitor of IKKs, Bay-117082 [51], or with a specific inhibitor of IKKβ, SC-514 [52]. Both inhibitors significantly suppressed the BZ-induced IL-8 mRNA expression in MDA-MB-231 cells (Fig 3A), indicating that the IL-8 expression induced by proteasome inhibition in TNBC cells is mediated by IKKβ. To confirm these data, we analyzed IL-8 mRNA expression and cytokine release in BZ-treated MDA-MB-231 cells transfected with IKKα, IKKβ, IKKε, or non-specific control siRNAs (Fig 3B). While transfection with IKKε did not have any significant effect on IL-8 mRNA levels (Fig 3C) or cytokine release (Fig 3D), transfection with IKKα, and particularly IKKβ, significantly suppressed the BZ-induced IL-8 expression (Fig 3C and 3D).

### BZ increases nuclear accumulation of p65 NFκB, and IKK-dependent p65 recruitment to IL-8 promoter in TNBC cells

**Fig 4:**
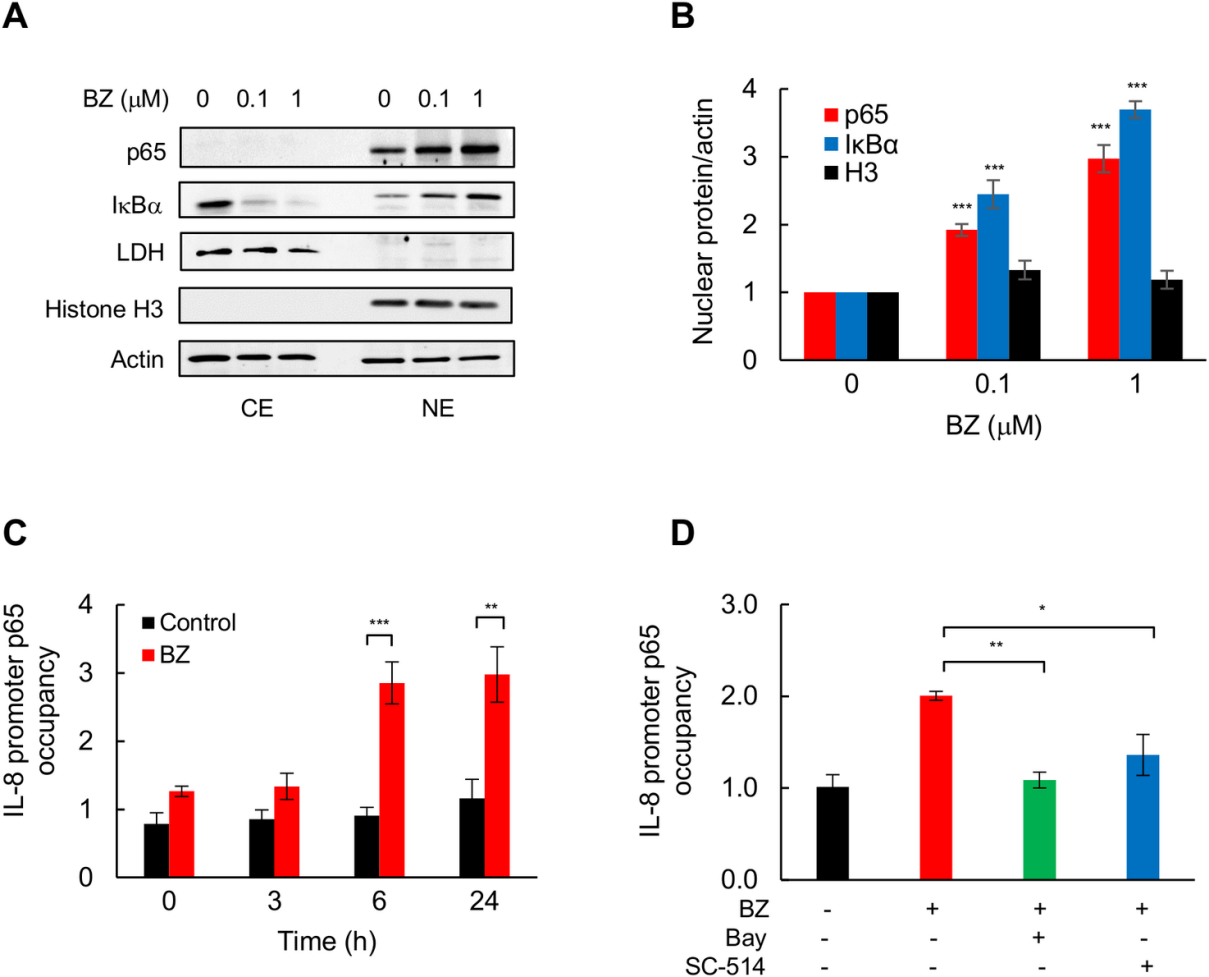
BZ increases nuclear p65 accumulation, and IKK-dependent p65 recruitment to IL-8 promoter in TNBC cells. **(A)** Western blotting of cytoplasmic (CE) and nuclear extracts (NE) prepared from MDA-MB-231 cells treated 24 h with BZ, and analyzed by using p65 and IκBα antibodies. The presence of cytoplasmic proteins in nuclear fraction was evaluated by re-probing the membrane with lactate dehydrogenase (LDH) antibody. Nuclear contamination in the cytoplasmic fraction was assessed using histone H3 specific antibody. To confirm equal protein loading, the membranes were stripped and re-probed with actin antibody. Each lane corresponds to approximately 5×10^4^ cells. **(B)** Densitometric evaluation of p65, IκBα, and histone H3 levels in nuclear extracts of BZ-treated MDA-MB-231 cells, shown in panel A. The densities of nuclear p65, IκBα, and histone H3 were normalized to the densities of nuclear actin. The values for untreated cells were arbitrarily set to 1, and the other values are presented relative to these values. **(C)** Recruitment of p65 to endogenous IL-8 promoter analyzed by ChIP and quantified by real time PCR in MDA-MB-231 cells treated with 100 nM BZ for 0, 3, 6 and 24 h. **(D)** ChIP of p65 recruitment to IL-8 promoter in MDA-MB-231 cells pre-incubated 12 h with control DMSO, Bay-117082 (10 μM), or SC-514 (10 μM), and then incubated 24 h with 100 BZ. The data are presented as the change in occupancy over the human IGX1A (Qiagen) sequence control and represent the mean +/− SE of three experiments. Asterisks denote a statistically significant change compared to control.

Because IL-8 transcription is regulated predominantly by p65 homodimers [19,34,35], we wanted to determine whether the increased IL-8 expression induced by proteasome inhibition in TNBC cells is associated with increased nuclear levels of p65 NFκB. Analysis of cytoplasmic and nuclear extracts by western blotting showed that p65 was localized predominantly in the nucleus of MDA-MB-231 cells (Fig 4A); this is consistent with the high constitutive NFκB activity in these cells [21–24]. Cell incubation with BZ further increased the nuclear accumulation of p65 (Fig 4A and 4B); this is likely caused by the BZ-mediated inhibition of p65 proteasomal degradation [37]. In addition, BZ induced nuclear translocation and accumulation of IκBα in MDA-MB-231 cells (Fig 4A and 4B), as was previously observed in other cancer cells [38–40]. However, since p65 homodimers exhibit a low affinity for IκBα [34,35], the nuclear accumulation of IκBα was not associated with the inhibition of IL-8 transcription in TNBC cells.

To determine whether proteasome inhibition increases IL-8 promoter occupancy by p65, we analyzed the kinetics of p65 promoter recruitment by chromatin immunoprecipitation (ChIP) in MDA-MB-231 cells treated with 100 nM BZ or control DMSO. As shown in Fig 4C, 6 h and 24 h incubation with BZ significantly increased p65 promoter occupancy, indicating that proteasome inhibition induces IL-8 transcription by increasing p65 promoter recruitment. To find out whether the BZ-induced p65 recruitment requires the kinase activity of IKK, MDA-MB-231 cells were pre-incubated with Bay-117082 or SC-514 before 24 h treatment with 100 nM BZ. Inhibition of IKK activity by Bay-117082 and SC-514 significantly attenuated the BZ-induced p65 occupancy at the IL-8 promoter (Fig 4D), indicating that IKK activity is required for the BZ-induced p65 recruitment to IL-8 promoter in TNBC cells.

### IKK inhibition enhances BZ cytotoxic and anti-proliferative effect in TNBC cells

**Fig 5:**
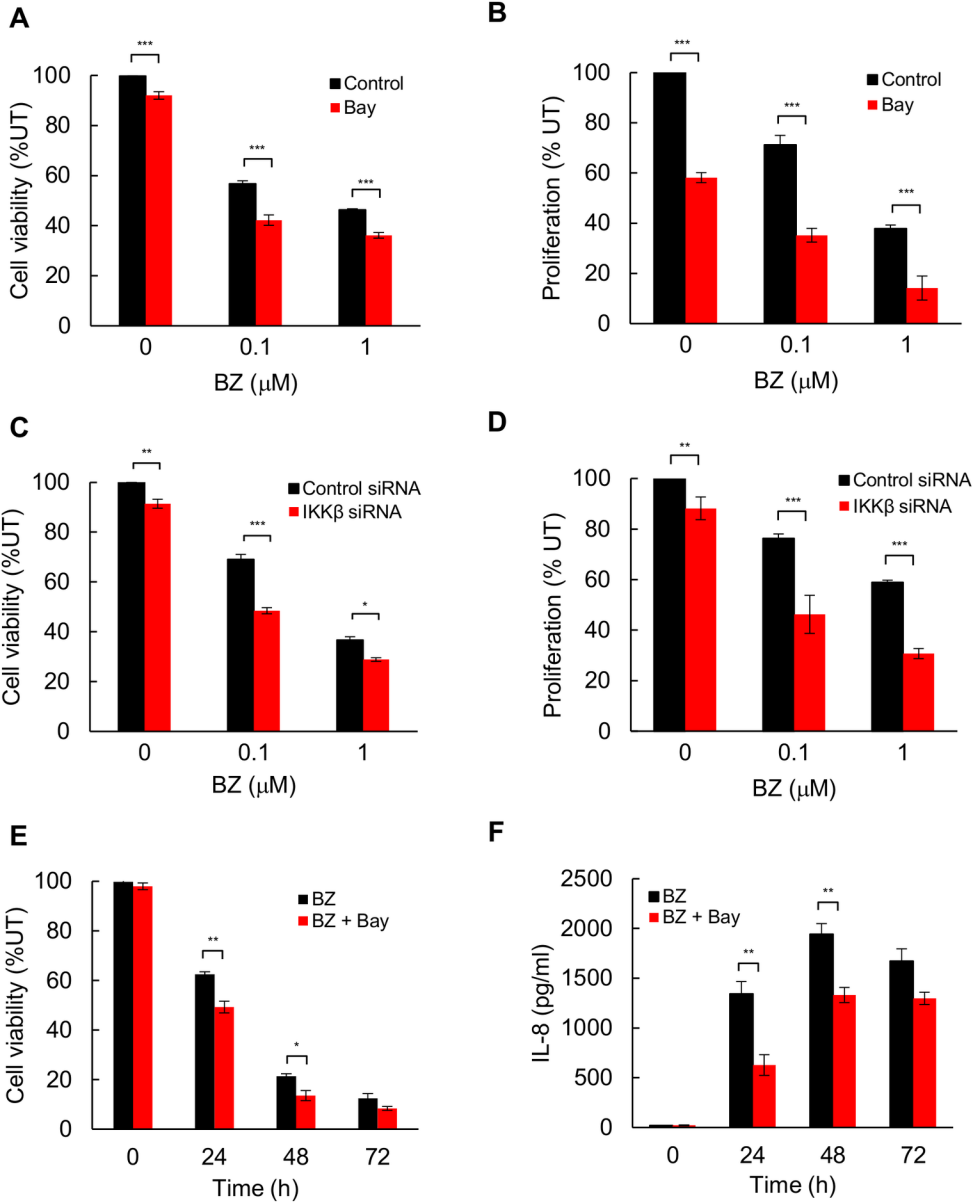
Suppression of IKK activity enhances BZ cytotoxic and anti-proliferative effect in TNBC cells. **(A)** Viability of MDA-MB-231 cells treated 24 h with BZ in the presence or absence of Bay-117082, analyzed by using Trypan Blue exclusion. **(B)** Proliferation of MDA-MB-231 cells treated 24 h with BZ in the presence or absence of Bay-117082, analyzed by CellTiter 96 One Solution Cell Proliferation Assay. **(C)** Viability of MDA-MB-231 cells transfected with control or IKKβ siRNA and incubated 24 h with BZ. **(D)** Proliferation of MDA-MB-231 cells transfected with control or IKKβ siRNA, and incubated 24 h with BZ. **(E)** Viability and **(F)** IL-8 release in MDA-MB-231 cells concomitantly incubated with 100 nM BZ with and without 10 μM Bay-117082 for up to 72 h. The data are expressed as the percentage compared to untreated cells. The values represent the mean +/−SE of four experiments; asterisks denote a statistically significant change compared to control.

Since our data indicated that the BZ-induced, IKK-dependent IL-8 release increases survival and proliferation of TNBC cells, we wanted to test whether inhibition of IKK activity would enhance the BZ cytotoxic and anti-proliferative effect. To this end, cell viability and proliferation were analyzed in MDA-MB-231 cells pre-incubated with the IKK inhibitor Bay-117082, and treated 24 h with increasing BZ concentrations. Inhibition of IKK activity by Bay-117082 significantly reduced cell viability (Fig 5A) and proliferation (Fig 5B) in BZ-treated cells. To confirm the above data by a pharmacologically independent approach, we analyzed cell viability and proliferation in BZ-treated MDA-MB-231 cells transfected with IKKβ siRNA or control non-specific siRNA. Compared to transfection with control siRNA, transfection with IKKβ siRNA significantly decreased viability (Fig 5C) and proliferation (Fig 5D) of BZ (24 h)-treated cells.

To determine whether inhibition of IKK activity enhances the BZ-cytotoxic effect beyond the 24 h time point, we analyzed cell viability and IL-8 release in MDA-MB-231 cells incubated with BZ with and without Bay-117082 for up to 72 hours. Even though the viability of MDA-MB-231 cells treated with BZ for 48 and 72 h was low, it was further decreased by the inhibition of IKK activity by Bay-117082 (Fig 5E), and this was associated with an inhibition of IL-8 release (Fig 5F).

### IKK and proteasome inhibitors have synergistic effect in reducing migration and invasion of TNBC cells

**Fig 6:**
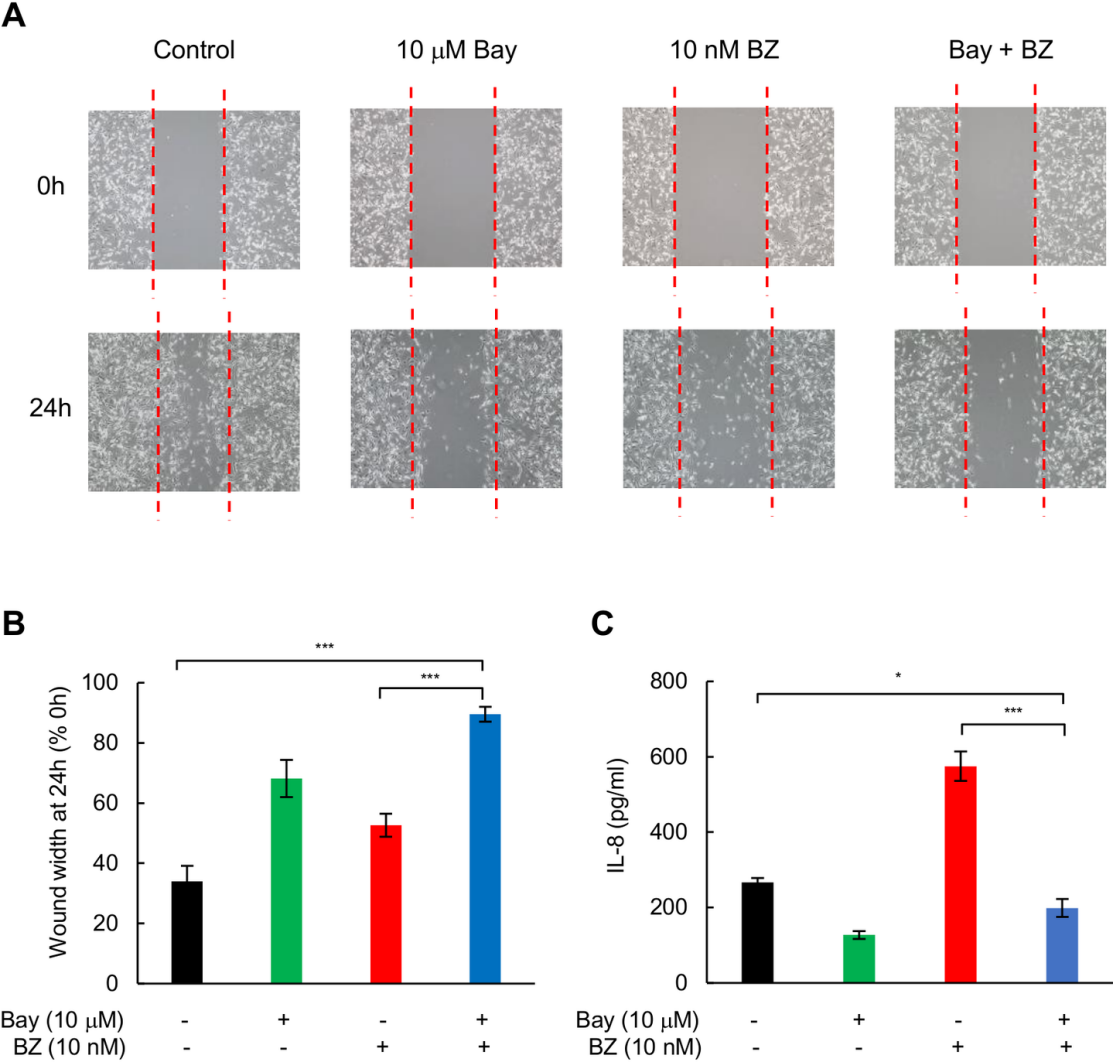
IKK and proteasome inhibitors have synergistic effect in reducing migration of TNBC cells. **(A)** MDA-MB-231 cells were incubated with 10 μM Bay-117082 and/or 10 nM BZ as indicated, and cell migration was evaluated by wound-healing assay as described in “Materials and methods”. **(B)** Images from panel A were quantified using ImageJ software, and the data are expressed as wound width at 24 h compared to the corresponding wound width at 0 h. **(C)** IL-8 release measured in cell supernatants in the wound-healing assay shown in panel A. The values represent the mean +/−SE of three experiments; asterisks denote a statistically significant change compared to control.

To extend these findings, we examined the effect of proteasome and IKK inhibition on migration and invasion of TNBC cells. Combination of 10 nM BZ and 10 μM Bay-117082 significantly decreased migration of MDA-MB-231 cells measured by the scratch wound-healing assay; both compared to control untreated cells, and compared to cells treated with BZ only (Fig 6A and 6B). In addition, combination of BZ and Bay-117082 significantly decreased the IL-8 release measured in the supernatants of scratched cells; both compared to untreated cells, and compared to cells treated with BZ alone (Fig 6C).

**Fig 7:**
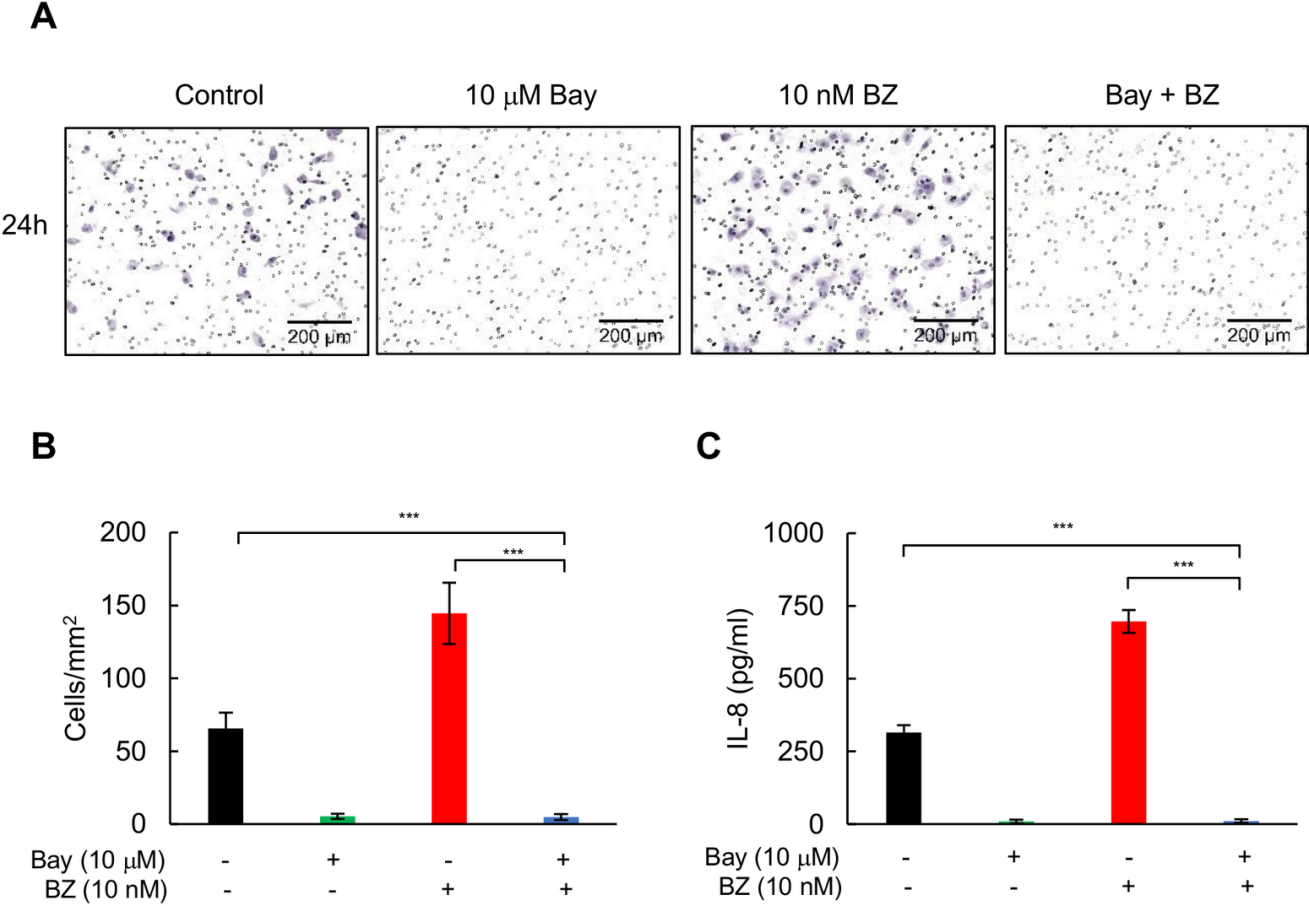
IKK inhibition decreases invasion of BZ-treated TNBC cells. **(A)** MDA-MB-231 cells were incubated with 10 μM Bay-117082 and/or 10 nM BZ as indicated, and cell invasion was measured as described in “Materials and methods”. **(B)** Quantification of images from panel A using ImageJ software. **(C)** IL-8 release measured in cell supernatants in the invasion assay shown in panel A. The values represent the mean +/−SE of three experiments; asterisks denote a statistically significant change compared to control.

Interestingly, 10 nM BZ induced Matrigel invasion of MDA-MB-231 cells (Fig 7A and 7B), and this was associated with increased IL-8 release by the cells (Fig 7C). IKK inhibition by Bay-117082 significantly decreased the invasion potential of BZ-treated cells (Fig 7A and 7B), and suppressed the BZ-induced IL-8 release (Fig 7C). Together, these data show that IKK inhibition significantly enhances the BZ inhibitory effect on viability, proliferation, and migration of TNBC cells, and inhibits the BZ-induced IL-8 expression and invasion.

## Discussion

Triple negative breast cancer comprises 15–20% of newly diagnosed breast cancer cases, and compared with other breast cancer subtypes, exhibits increased aggressiveness and less favorable prognosis. Since TNBC patients are unresponsive to current targeted therapies, new therapeutic strategies are vital. Although previous studies have shown that proteasome inhibition induces apoptosis in TNBC cells [49,50], suggesting a potential therapeutic activity in TNBC, clinical trials using PI as single agents have produced disappointing results. In this study, we demonstrate that proteasome inhibition induces expression of IL-8 and its receptors CXCR1 and CXCR2, resulting in increased survival, proliferation, and migration of TNBC cells. The induced IL-8 expression is mediated by IKK, and associated with an increased nuclear accumulation of p65 NFκB, and IKK-dependent p65 recruitment to IL-8 promoter. Inhibition of IKK activity significantly decreases proliferation, migration, and invasion of BZ-treated TNBC cells. These results are the first to show that proteasome inhibition increases IL-8 signaling in TNBC cells, indicating that targeting IKK activity might increase PI effectiveness in the treatment of TNBC.

Hundreds of genes are targeted by proteasome, suggesting that proteasome inhibition might not be compatible with normal cellular functions. Interestingly, a number of studies have demonstrated an increased susceptibility of transformed cancer cells, compared to untransformed cells, to proteasome inhibition. While the exact molecular basis for this increased sensitivity remains incompletely understood, several studies have shown that rapidly dividing and proliferating cells are more susceptible to PI-induced apoptosis than quiescent cells [53]. Since NFκB signaling is increased in cancer cells, and promotes their proliferation and migration, it likely represents one of the main targets of PI in cancer cells, compared to normal cells.

Interestingly, however, from the tested NFκB-regulated genes, only expression of IL-8, and its receptors CXCR1 and CXCR2, was induced by proteasome inhibition in TNBC cells (Figs 1A and 2A), demonstrating that the regulation of NFκB-dependent transcription by PI is gene specific. What are the mechanisms regulating specificity of NFκB-dependent transcription in response to proteasome inhibition? Both IκBα and p65 can serve as proteasome substrates [8–10,33]. However, while IκBα stabilization by PI downregulates NFκB-dependent transcription, p65 stabilization has the opposite effect, and may increase transcription of p65-regulated genes [54,55], especially genes regulated by p65 homodimers. Thus, the effect of PI on NFκB-dependent transcription likely depends on the subunit composition of NFκB complexes at the corresponding promoters. Unlike other NFκB-dependent genes, the IL-8 transcription is regulated predominantly by NFκB p65 homodimers that have a low affinity for IκBα, resulting in the relative resistance to inhibition by nuclear IκBα [34,35]. Therefore, even though proteasome inhibition stabilizes IκBα and increases its nuclear accumulation, resulting in the inhibition of genes regulated by NFκB p65/50 heterodimers, it concomitantly induces expression of IL-8, and perhaps other NFκB-dependent genes that are regulated by p65 homodimers. In this regard, it will be interesting to determine in the future whether transcription of CXCR1 and CXCR2, which are also increased by BZ in TNBC cells (Fig 2), is also regulated by p65 homodimers.

Importantly, our results demonstrate that the proteasome inhibition-induced IL-8 expression in TNBC cells is dependent on IKK activity, and indicate that it is mediated by IKKα and IKKβ, but not IKKε (Fig 3). Despite the limited effectiveness of PI and IKK inhibitors as single agents in the treatment of TNBC and other solid tumors, accumulating evidence indicates that combining PI and IKK inhibitors increases their cytotoxic effect in solid tumors [23,39,40,56,57]. Combination of BZ and an IKK inhibitory peptide had a synergistic effect on inhibiting proliferation of ER-negative breast cancer MDA-MB453 cells [23]. Inhibition of IKKβ activity also increased the BZ cytotoxic effect in ER-positive breast cancer MCF-7 and T47D cells [56]. Furthermore, IKK inhibition increased effectiveness of BZ in reducing ovarian tumor growth *in vivo* [57].

Our results indicate that the mechanistic basis of this synergy consists of the induced, IKK-dependent expression of IL-8, which increases survival, proliferation, and migration of solid cancer cells. However, it is important to note that our study was done *in vitro*, using three TNBC cell lines, MDA-MB-231, MDA-MB-468, and HCC-1937. These cell lines are basal-like cells, characterized by increased proliferation and invasiveness compared to the ER/PR-positive MCF-7 cells, and mutated tumor suppressor p53 [58–60]. In addition, compared to the ER/PR-positive MCF-7 cells, these TNBC cells are characterized by increased constitutive expression of IL-8, which has been associated with the increased invasive phenotype of these cells [3,4,6]. However, even though these cells have been widely used as an *in vitro* model to study TNBC, they lack proper tumor microenvironment formed by tumor cells, immune cells, and the surrounding tumor stroma [61]. Thus, future studies should determine whether IKK inhibition increases effectiveness of PI in TNBC animal models, and ultimately in TNBC patients.

## Conclusions

Together, our results show that proteasome inhibition upregulates IL-8 signaling in TNBC cells. Suppression or neutralization of the BZ-induced IL-8 potentiates the BZ cytotoxic and anti-proliferative effect. The IL-8 expression induced by proteasome inhibition is mediated by IKK, and inhibition of IKK activity decreases proliferation, migration, and invasion of BZ-treated TNBC cells. These data provide a mechanistic rationale for clinical studies using proteasome inhibitors in combination with IKK inhibitors in the treatment of TNBC.
